# Estimating the full health and economic benefits of current and future influenza vaccines

**DOI:** 10.1186/s12916-023-02995-4

**Published:** 2023-07-27

**Authors:** K. E. Lafond, R. Gharpure, V. G. Dugan, E. Azziz-Baumgartner

**Affiliations:** Influenza Division, Centers for Disease Control and Prevention, Atlanta, GA 30307 USA

**Keywords:** Influenza, Global health, Vaccines, Burden of disease, Cost-effectiveness

## Abstract

In the dynamic landscape of respiratory virus vaccines, it is crucial to assess the value of novel mRNA and combination influenza/COVID-19 vaccines in low- and middle-income countries. Modeling studies, such as the one conducted by Waterlow et al., provide vital information about the cost–benefit potential of these products compared to currently licensed vaccines. However, this approach only accounts for directly measured medically attended influenza-associated illnesses and has two major limitations. First, this method fails to capture the full disease burden of influenza (including non-respiratory and non-medically attended influenza illnesses), which are particularly important drivers of disease burden in infants and older adults. Second, the model does not describe the ancillary benefits of influenza vaccination such as the attenuation of severe disease, prevention of severe non-respiratory outcomes (e.g., myocardial infarctions), or reduced antibiotic use. To obtain a comprehensive understanding of the benefits of influenza vaccines, we must strive to improve the inputs for future modeling-based evaluations.

The global influenza vaccine landscape is dynamic, with novel products such as messenger ribonucleic acid (mRNA) and combination influenza/coronavirus disease 2019 (COVID-19) vaccines currently being pursued. The recent publication by Waterlow et al. is an important contribution to the emerging evidence about the marginal benefit of these novel products when compared to currently used influenza vaccines. While such analyses are useful, it is important for readers to understand their limitations. Sentinel surveillance systems, often used to generate disease and cost inputs for economic modeling, can substantially underestimate the true value of influenza and the benefit of vaccination. To fully understand the benefit of influenza vaccines, especially in low- and middle-income countries, we need to work toward better inputs in at least three aspects of future full value proposition models.

First, the use of hospital-based surveillance for severe acute respiratory infections (SARI) as a primary measure of the burden of influenza disease overlooks two important categories of influenza-associated illness: non-SARI hospitalizations and non-hospitalized severe disease. While the SARI case definition was designed to efficiently identify influenza viruses at a minimum cost to surveillance systems, it lacks sensitivity in capturing the full spectrum of severe influenza-associated illness [1, 2]. This is particularly the case among infants and older adults who often have non-respiratory clinical presentations or complications related to influenza. In one multi-country cohort, respiratory signs and symptoms were shown to underestimate influenza-associated hospitalizations among infants by a factor of 2.6 [3]. Older adults with influenza are also frequently hospitalized for the management of cardiac, endocrine, or other non-respiratory complications precipitated by influenza illnesses [4]. Moreover, individuals at high risk for severe disease, including older adults, may avoid seeking medical care and therefore remain undetected by routine surveillance systems. One large cohort in India recently found that 90% of adults 60 years and older with lower respiratory tract illnesses did not seek any medical attention (unpublished data from Indian Network of Population-Based Surveillance Platforms for Influenza and Other Respiratory Viruses among the Elderly (INSPIRE) cohort [5]).

Secondly, simple inputs of vaccine effectiveness (VE) tend to underestimate the direct benefits that vaccines offer to individuals, such as reducing the severity of influenza symptoms or preventing non-respiratory complications. Notably, averted illness and cost-effectiveness models do not typically account for vaccine-mitigated influenza illness. Findings from multi-year, multi-country VE networks demonstrate that those who are vaccinated against influenza but nevertheless develop influenza illness, remain in the hospital for fewer days, are less likely to require intensive care unit (ICU) admission, or die while hospitalized as a result of their illness [6]. Similarly, cost-effectiveness models do not typically account for the benefits of vaccines in preventing non-respiratory influenza complications. For example, pregnant women vaccinated against influenza have a lower risk of influenza illness and adverse birth outcomes during the influenza season [7] and give birth to infants who are at a lower risk of influenza infection compared to unvaccinated pregnant women [8].

Lastly, it is important to consider the broader benefits of influenza vaccination beyond just individual-level protection. One important but indirect benefit of vaccination is that prevention of medically attended influenza illnesses helps preserve healthcare resources during epidemics and reduces the use of antibiotics as a first-line treatment. These vaccine-derived benefits are frequently cost-effective or cost-saving [9] even when they do not account for prevention of inappropriate antibiotic use; community-level reductions in antimicrobial resistance could also be quantified [10] and might demonstrate increased cost-effectiveness. Additional community-level impacts may go beyond the capacities of standard modeling approaches, such as potential advances in health equity. For instance, vaccination may prevent lower-income households from facing catastrophic health expenses.

To adequately describe the value of current trivalent, quadrivalent, and enhanced influenza vaccines, and properly compare these with newer, yet to be licensed (e.g., mRNA) vaccine products for influenza, we need to leverage our shared knowledge on the full burden of influenza and the benefits of vaccination. This involves starting with standard surveillance outcomes [11] and also accounting for broader measures of disease burden and vaccine-averted illness [12]. By expanding this evidence base, we can make better-informed policies and investments that lead to better global health outcomes [13].

## Data Availability

No data was associated with this article.

## Abbreviations

COVID-19: Coronavirus disease 2019
ICU: Intensive care unit
INSPIRE: Indian Network of Population-Based Surveillance Platforms for Influenza and Other Respiratory Viruses among the Elderly
mRNA: Messenger ribonucleic acid
SARI: Severe acute respiratory infections
VE: Vaccine effectiveness

## References

[1] Gordon A, Reingold A (2018). The burden of influenza: a complex problem. Curr Epidemiol Rep.

[2] Macias AE (2021). The disease burden of influenza beyond respiratory illness. Vaccine.

[3] Thompson MG (2019). Underdetection of laboratory-confirmed influenza-associated hospital admissions among infants: a multicentre, prospective study. Lancet Child Adolesc Health.

[4] Chow EJ, et al. Acute cardiovascular events associated with influenza in hospitalized adults : a cross-sectional study. Ann Intern Med. 2020;173(8):605–13. 10.7326/M20-1509 PMC8097760 32833488

[5] Krishnan A (2021). Cohort profile: Indian Network of Population-Based Surveillance Platforms for Influenza and Other Respiratory Viruses among the Elderly (INSPIRE). BMJ Open.

[6] Regan A, et al. Severity of influenza illness associated with seasonal influenza vaccination among hospitalized patients in four South American countries, 2013–2019: a surveillance-based cohort study. Lancet ID. 2023;23(2):222–32. 10.1016/S1473-3099(22)00493-5 PMC9876808 36206790

[7] Duque J (2023). Multi-decade national cohort identifies adverse pregnancy and birth outcomes associated with acute respiratory illness hospitalisations during the influenza season. Influenza Other Respir Viruses.

[8] Azziz-Baumgartner E, Grohskopf L, Patel M (2021). Realizing the potential of maternal influenza vaccination. JAMA.

[9] Peasah SK (2013). Influenza cost and cost-effectiveness studies globally–a review. Vaccine.

[10] Smith ER (2020). Reducing antibiotic use in ambulatory care through influenza vaccination. Clin Infect Dis.

[11] McCarron M (2022). United States Centers for Disease Control and Prevention support for influenza surveillance, 2013–2021. Bull World Health Organ.

[12] Roguski KM (2020). Variability in published rates of influenza-associated hospitalizations: a systematic review, 2007–2018. J Glob Health.

[13] Jit M, Newall AT, Beutels P (2013). Key issues for estimating the impact and cost-effectiveness of seasonal influenza vaccination strategies. Hum Vaccin Immunother.

